# Complete Genome Sequence of *Southern tomato virus* Identified in China Using Next-Generation Sequencing

**DOI:** 10.1128/genomeA.01226-15

**Published:** 2015-10-22

**Authors:** Chellappan Padmanabhan, Yi Zheng, Rugang Li, Shu-E Sun, Deyong Zhang, Yong Liu, Zhangjun Fei, Kai-Shu Ling

**Affiliations:** a USDA-Agricultural Research Service, U.S. Vegetable Laboratory, Charleston, South Carolina, USA; b Boyce Thompson Institute for Plant Research, Cornell University, Ithaca, New York, USA; c Hunan Plant Protection Institute, Hunan Academy of Agricultural Sciences, Changsha, Hunan, China; d USDA-Agricultural Research Service, Robert W. Holley Center for Agriculture and Health, Ithaca, New York, USA

## Abstract

The complete genome sequence of *Southern tomato virus* (STV), a double-stranded RNA virus that affects tomato in China, was determined using small RNA deep sequencing. This Chinese isolate shares 99% sequence identity to other isolates from Mexico, France, Spain, and the United States. This is the first report of STV infecting tomatoes in Asia.

## GENOME ANNOUNCEMENT

*Southern tomato virus* (STV), which causes tomato mosaic and yellow stunting disorder, was first characterized for its presence in Mexico and the United States (1). Recently, it has also been identified in France (2) and Spain (3). STV is a double-stranded RNA (dsRNA) virus with a small genome of approximately 3.5 kb (1). Its dsRNA genome and unique genome organization place it between the *Totiviridae* and *Partitiviridae* families, in the genus *Amalgavirus* of the family *Amalgaviridae* (1). STV appears to have a high rate of seed transmission, but with no apparent evidence of graft or mechanical transmission (1). However, its presence in commercial seeds around the world warrants further investigation. 

In the summer of 2012, tomato plants in a greenhouse near Shouguang, Shandong province, in eastern China, exhibited a high incidence of virus-like disease symptoms, with severe mosaic, epinasty, leaf curl, and yellow stunting disorder. To determine the causal agent(s), total RNA was prepared with TRIzol reagent on a pooled sample collected from eight diseased tomato plants. The small RNA deep-sequencing technology (4) was employed for virus identification. A small RNA library was prepared as described previously (5) and sequenced using an Illumina HiSeq 2000. To identify possible viruses, sRNA sequences were assembled and analyzed accordingly (6). From the preliminary sequence assemblies and analyses, in addition to a full-genome sequence of STV, several other viruses, including *Cucumber mosaic virus* (CMV), *Tomato yellow leaf curl virus* (TYLCV), and *Tomato chlorosis virus* (ToCV) were also identified. With such a high incidence of mixed infection, the disease symptoms observed could not be attributed to STV alone. For STV, the complete genome in a single contig was obtained, and its sequence was verified by Sanger sequencing through genome walking using reverse-transcription PCR with five pairs of STV-specific primers. 

The verified complete genome for the Chinese isolate STV_CN12 comprised 3,463 nucleotides (GenBank accession no. KT438549). The genome contained a 5′-proximal open reading frame (ORF) encoding a 378–amino acid (aa) coat protein (p42). A second ORF contained an RNA-dependent RNA polymerase (1,063 aa), which was likely expressed via an a + 1 ribosomal frameshift, as predicted for other STV isolates (1). BLASTn searches to the NCBI databases revealed that STV_CN12 shared 99% nucleotide sequence identities with other STV isolates identified from Mexico (EF442780), the United States (EU413670), France (KC333078), and likely Spain (3). STV is a seed-transmitted virus. With such strong sequence conservation among the known STV isolates, they may share an origin, likely from contaminated seeds. To our knowledge, the identification of a tomato-infecting isolate (STV_CN12) in China was the first report of STV in Asia, which is an important hybrid tomato seed–producing region for a number of seed companies. Therefore, additional surveys and characterization to their biological and molecular properties would be necessary. With seed transmission as the only known pathway for STV, using a certified virus-tested seed should be considered for disease management.

### Nucleotide sequence accession number.

The nucleotide sequences of STV_CN-12 have been deposited in GenBank under the accession number KT438549.
